# The role of Periodic Safety Update Reports in the safety management of biopharmaceuticals

**DOI:** 10.1007/s00228-012-1317-3

**Published:** 2012-06-17

**Authors:** Hans C. Ebbers, Aukje K. Mantel-Teeuwisse, Fakhredin A. Sayed-Tabatabaei, Ellen H. M. Moors, Huub Schellekens, Hubert G. M. Leufkens

**Affiliations:** 1 Division of Pharmacoepidemiology and Clinical Pharmacology, Utrecht Institute for Pharmaceutical Sciences (UIPS), Faculty of Science, Utrecht University, P.O. Box 80 082, 3508 TB Utrecht, The Netherlands; 2 Medicines Evaluation Board, Utrecht, The Netherlands; 3 Innovation Studies, Copernicus Institute of Sustainable Development, Utrecht University, Utrecht, The Netherlands; 4 Department of Pharmaceutics, Utrecht Institute for Pharmaceutical Sciences (UIPS), Faculty of Science, Utrecht, The Netherlands

**Keywords:** Periodic Safety Update Report, Pharmacovigilance, Biopharmaceuticals, Safety

## Abstract

**Purpose:**

To describe and assess the outcomes of Periodic Safety Update Report (PSUR) evaluations of biopharmaceuticals.

**Methods:**

A cross-sectional analysis was performed of follow-up requirements of PSURs submitted for centrally approved biopharmaceuticals in the European Union between 1 July 2008 and 30 June 2010. A follow-up analysis on a subset of products that submitted multiple PSURs within the study period was also performed.

**Results:**

The cross-sectional analysis included 70 PSURs. Potential safety concerns occurred in 57 (83 %) of all PSURs, and 26 (37 %) concluded a need to change the Summary of Product Characteristics (SPC). In comparison to newer products, products authorized for more than 10 years contained significantly fewer potential safety concerns (60 vs. 92 %; *p* < 0.01) and required fewer SPC changes (15 vs. 46 %; *p* = 0.03). For 45 products, multiple PSURs were submitted that could be included in a follow-up analysis. For this subset of products, of the 106 newly identified safety potential safety issues, 7 (7%) resulted in requirements for label changes in the following PSUR.

**Conclusions:**

PSURs facilitate communication between regulators and marketing authorization holders. Potential safety concerns occur for the majority of biopharmaceuticals and throughout their lifecycle, but for established products PSUR evaluations rarely lead to regulatory actions.

## Introduction

Marketing authorization holders (MAHs) have the obligation to monitor the safety of their products after their product receives marketing authorization. As such, they are engaged in continuous dialogue with regulators to ensure that the right strategies are employed to optimize the benefit to risk ratio of their products. One of the main tools used to facilitate post-authorization communication between MAHs and regulators is the Periodic Safety Update Report (PSUR). PSURs aim to provide an update of worldwide safety experience with a specific pharmaceutical. It includes data from spontaneous reports, safety data from interventional and/or observational studies, as well as other relevant safety information (Table 1) [1]. PSURs are intended to proactively present, analyze, and evaluate new or changing safety data from any source evaluated in relation to estimates of exposure to the product, although total coverage of data sources may have limitations in practice [2]. PSURs are composed by MAHs and submitted to regulatory authorities for assessment at predetermined time points [1]. In the European Union (EU), PSURs also need to be submitted alongside applications to renew the initial marketing authorization, which is valid for a period of 5 years. Both regulatory authorities and MAHs spend significant resources on the creation and assessment of PSURs [3]. However, the outcomes of these efforts have not been well described.

**Table 1:** Structure and content of a Periodic Safety Update Report

Structural components of a PSUR	Description
Title page	
Executive Summary	
Introduction	Includes information on the product(s) included in the PSUR and placed into perspective of previous PSURs
Worldwide market authorization status	An overview of all the countries where the product is authorized, including differences in qualifications or indications
Update of regulatory authority or MAH actions taken for safety reasons	Worldwide Actions relating to safety that were taken during the period covered by the PSUR (or between data lock point and PSUR submission).
Changes to reference product information	Changes such as contraindications, precautions, warnings, adverse reactions, or interactions already made during the PSUR period should be described.
Patient exposure	An estimate of the patient exposure during the PSUR period and the method used to derive this estimate
Presentation of individual case histories	A description and analysis of selected cases, including fatalities, presenting new and relevant safety information. Includes a discussion of spontaneous reports, literature reports, reports originating from PASS or consumer reports
Studies	Discussion of data from studies that could potentially impact the product information. This includes newly analyzed company sponsored studies, targeted safety studies examining specific safety concerns and published studies.
Other information	Discussion on efficacy-related information, late breaking information and a discussion of the risk management plan
Overall safety evaluation	A concise discussion on all the data presented highlighting changes in both listed and unlisted adverse events
Conclusion	Assessed of all data in relation to the reference safety information and recommended action to be taken
Appendices	Including the Company Core Data Sheet, detailed information on ADRs in line listings and summary tabulations.

PSUR, Periodic Safety Update Report; MAH, marketing authorization holder; ADRs, adverse drug reactions

The concept of PSUR reporting in its current form stems from 1992 [4]. It has been noted at several ‘platforms’, including the International Conference of Harmonization (ICH) and the EU, that PSUR reporting has not kept pace with developments in pharmacovigilance, such as electronic adverse event reporting and risk management planning [5–7]. In 2010, this awareness resulted in changes in European legislation laying down the requirements for PSUR reporting [8]. In an earlier study on the determinants of safety-related regulatory actions for biopharmaceuticals, we found that PSUR evaluations contributed to 38 % of post-authorization regulatory actions in a sample of biopharmaceuticals [9]. In addition, in 2010, Alvarez et al. found that 64 % of a selection of adverse drug reactions (ADRs) originated from PSURs [10]. Both these studies examined the contribution of PSURs to identified safety signals, which does not provide insights as to *how* PSURs contribute to monitoring safety, or which fraction of PSURs leads to regulatory action. Multiple factors, including product characteristics, regulatory approval status and timing of approval could potentially affect the outcome of PSUR evaluations. Therefore, this study aims to address two topics: (1) to evaluate the outcomes of PSUR evaluations and identify determinants for PSURs that lead to regulatory actions, defined as safety-related changes, to the product labeling; (2) to assess the outcomes of safety-related follow-up requirements that resulted from PSUR assessment. Several recent studies have reported on the specifics of pharmacovigilance for biopharmaceuticals. The nature of reported adverse events for biopharmaceuticals seems to differ from those for small molecules, which may lead to different safety-related regulatory actions and could necessitate a distinctive pharmacovigilance approach [11, 12]. To add to this work and to increase the understanding of the performance of pharmacovigilance activities in the safety management of these products, we have limited this study to biopharmaceuticals.

## Methods

### Study design

A cross-sectional analysis was performed of all PSURs and PSUR assessment reports (PARs) created for biopharmaceuticals centrally approved in the EU since 1995. Biopharmaceuticals are defined as therapeutic proteins with active agents inherently biological in nature and manufactured using biotechnology, including recombinant therapeutic proteins (including antibodies), nucleic-acid based products and engineered cell or tissue-based products [13]. For the purpose of our study we excluded vaccines, diagnostics and extracted products that are not manufactured using biotechnology (e.g. proteins and/or blood products extracted from non-engineered sources).

**Fig. 1:**
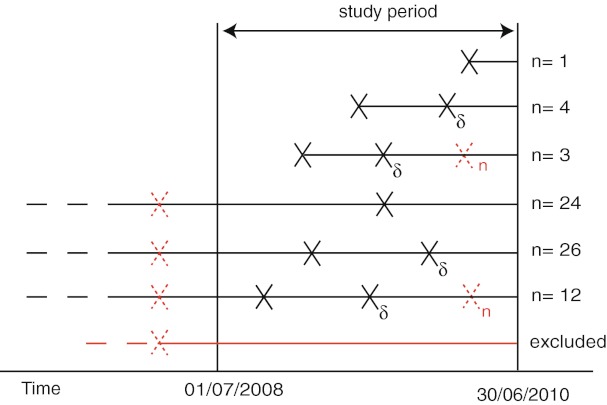
Graphical representation of the possible Periodic Safety Update Reports (PSURs) included in the study. *X* PSURs included in the main analysis, *Xδ* PSURs included in the follow–up analysis. Each third or more PSUR submitted within the study period was excluded, as indicated by the *dashed X*_*n*_

PSURs and PARs were obtained from the repository of the Dutch Medicines Evaluation Board (CBG-MEB). As PSURs contain proprietary information, the data were confidentially collected and analyzed in an aggregated fashion. For the main analysis, each first PSUR submitted for each product in a recent timeframe (1 July 2008 to 30 June 2010) was included (Fig. 1). Because of the intrinsic limitations of the electronic repository, only these more recent PSURs could be included, which limited the long-term follow-up of individual products. Authorization details of the products were obtained from European Public Assessment Reports (EPARs), which are available from the website of the European Medicines Agency (EMA) [14]. The position of the biopharmaceutical in the Anatomic Therapeutic Classification (ATC) was determined using the website of the World Health Organization (WHO) Collaborating Center for Drug Statistics Methodology [15]. Authorization times were based on the international birth date, which is the first date of marketing authorization anywhere in the world. All other product-related information was obtained from the PSURs and PARs.

### Scoring procedure

PSURs and PARs were analyzed for follow-up ‘requirements’, which included both commitments proposed by the MAH and requirements from the assessor at the regulatory authority. If these conflicted, the requirement within the PAR was included in the analysis. We created a scoring method analogous to the safety specification of Risk Management Plans in which we categorized follow-up requirements as ‘identified risks’, ‘potential risks’ and ‘presentation of risk data’ [1] (Box 1). In addition, categories for quality-related issues and other, non-safety-related follow-up requirements were created.

**Box 1:** Scoring procedure of follow-up requirements. Safety concerns were scored once in each category

1. Identified Risks	a. Requirements to change to the current Summary of Product Characteristics (SPC).
2. Potential risks (Safety-related follow-up requirements)	a. Provision of a review of all data on a possible safety concern, either in the next PSUR or before the next PSUR (‘cumulative review’).
	b. Requirements to closely monitor cases of new (suspected) safety concerns in forthcoming PSURs. Such concerns are discussed in detail in each PSUR.
	c. The continued close monitoring of safety concerns that had been previously identified, this included all items that were described as ‘under heightened surveillance’.
	d. Instances when it was explicitly mentioned that a previous requirement for continuous monitoring was no longer required, this includes statements that a previously identified safety concern is now subject to ‘routine monitoring’.
	e. Requirements to provide additional information on a possible safety concern that did not require a cumulative review of data or the continued monitoring were included in this category. This includes requirements to comment on individual cases, studies included in the PSUR or on the results of a previously presented cumulative review.
3. Presentation of risk data	a. Requirements to provide information that was missing in the current PSUR, e.g. missing analyses not related to a specific safety concern. For example, results of a safety study that were not discussed, or missing estimates of patient exposure.
	b. Requirements to present the information in another way to facilitate the assessment of the PSUR. Examples of this category were requirements to split data for different approved indications or presenting data for patient sub groups.
4. Quality related follow-up requirements.	a. Clarification of discrepancies within the PSUR (e.g. between tables and texts, or between tables and case reports).
	b. Requirements for improving the quality and/or follow-up of individual case reports were included in this category.
5. All other follow-up requirements	a. This included requirements unrelated to the safe use of the product, comments on the provision of study reports, remarks that did not require follow-up, updates of ongoing regulatory procedures, changes in SPC wording of safety issues that were identified before the current PSUR assessment pro

When a single safety concern included multiple follow-up requirements to address the potential risks (category 2), each was scored once according to the following ‘hierarchy’: cumulative reviews > close monitoring of a potential new safety concern > continued close monitoring of a potential safety concern > additional information on a possible safety concern. For example, if for a given product that was being closely monitored a cumulative review was required, this was scored only once as a cumulative review. All frequencies were recorded and recoded into dichotomous (Y/N) variables for the main analysis. The dataset was scored by a single rater (HE). To test the reliability of the scoring procedure, 14 randomly selected products, including 214 items, were scored by two independent assessors (HE and MP), disagreement was resolved by consensus. Agreement between initial scoring and consensus was good (κ = 0.93) on individual items. In 5 of 14 products this led to an altered overall score in one or more of the 11 dichotomous variables. The results of which were include in the final dataset.

### Follow-up analysis of the outcome of new potential safety concerns

For the subset of products for which two or more PSURs were submitted within the study period, we performed an analysis of follow-up requirements (Fig. 1). We assessed the second of two consecutive PSURs for co-occurrence of events that were scored as new potential safety issues in the first PSUR. New potential safety issues were defined as all safety concerns in the first PSUR which resulted in requirements for either a ‘cumulative review’ or ‘close monitoring of potential new safety concern’.

### Data analysis

All data were entered into a database and analyzed using SPSS Statistics software package, ver. 19.0.0 (SPSS, Chicago, IL). Associations between requirements for Summary of Product Characteristics (SPC) changes and determinants, including product characteristics, regulatory authorization characteristics and time since approval, were tested using two-sided Fisher’s exact tests at an α level of 0.05. Ratios were calculated using Epi Info^TM^ v. 7.0.8.0 [Centers for Disease Control and Prevention (CDC), Atlanta, GA].

## Results

### Summary characteristics

We identified 115 biopharmaceutical products that were approved during the study period. Of these, 33 did not submit PSURs during the study period, and 12 were excluded because multiple products were included in a single PSUR (all fast- and intermediate-acting insulins and biosimilars sold under different trade names). Therefore, the final analysis included 70 PSURs (Table 2). The median time between authorization and the date of submission of the first included PSUR was 6.9 (range 0.6–24.6) years. Monoclonal antibodies and hormones accounted for over half of the products in the sample. Most products belonged to the ATC groups of antineoplastic and immunomodulating agents (*n* = 26), blood and blood-forming organs (*n* = 16) and alimentary tract and metabolism (*n* = 13).

**Table 2:** Key characteristics of the products included in the cross-sectional study

Key characteristics	*n*	%
Total	70	100
Regulatory approval characteristics		
Approved under exceptional circumstances	2	2.9
Orphan status	3	4.3
Both exceptional circumstances and orphan status	5	7.1
Regular approval	60	85.7
First in ATC Class		
Yes	11	15.7
No	53	75.7
Biosimilars^a^	6	8.6
Mechanistic class		
Enzymes	8	11.4
Growth factors	8	11.4
Hormones	18	25.7
Interferons	6	8.6
Monoclonal antibodies	19	27.1
Recombinant blood products	6	8.6
Receptors	2	2.9
Others/various	3	1.3
ATC class		
A: Alimentary tract and metabolism	13	18.6
B: Blood and blood forming organs	16	22.9
H: Systemic hormonal preparations, excl. sex hormones and insulins	6	8.6
L: Antineoplastic and immunomodulating agents	26	37.1
M: Musculo-skeletal system	4	5.7
R: Respiratory system	1	1.4
S: Sensory organs	1	1.4
V: Various	3	4.3

ATC, Anatomic Therapeutic Classification

^a^For several biosimilar products ‘Joint PSURs’ were created that contained safety information of the same molecule marketed under various names. 1: Abseamed, binocrit and epoetin hexal; 2: filgrastim hexal and Zarzio; 3: tevagrastim, biograstim, ratiograstim and filgrastim ratiopharm

The characteristics of PSURs varied considerably for the different products. The median length of PSURs was 396 (range 15–30,901) pages. Excluding the appendices that contained detailed information on individual cases in so-called ‘line listings’, the median length of a PSUR was 69 (range 9–949) pages. The median length of a PAR was 17 (range 2–82) pages. Each PSUR contained a median of 305 case reports; some reports included over 12,000 case reports, describing over 20,000 adverse events.

### Cross-sectional analysis of PSUR assessments

**Fig. 2:**
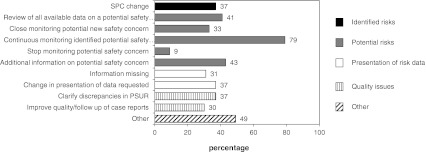
Outcome of PSUR assessments. Percentage of PSUR evaluations that included at least one of the outcomes

Identified safety concerns, such as changes to the SPC, were included in 27 (39 %) of the PSURs. In one case, this required communication to healthcare professionals through a so-called Direct Healthcare Professional Communication. Potential safety concerns were present in 58 (83 %) of the PSURs (Fig. 2). New potential safety concerns were identified in 23 (33 %) of the PSURs, and 55 (79 %) of all the assessments resulted in the continued monitoring of a previously identified safety concern. The number of concerns that were closely monitored ranged from one to 34 concerns per PSUR. For 46 (66 %) of the products, either changes to the presentation of risk data were required or quality issues were identified. Five (7 %) of the PSURs did not result in any follow-up requirement. Excluding PSURs that were submitted for products that were suspended at the time of submission (*n* = 2), none of the PARs concluded that the overall benefit/risk (B/R) balance had change—for all assessed products the benefits continued to outweigh the risks.

The proportion of PSUR assessments that led to proposals for SPC changes in relation to various subgroups is shown in Table 3. Requirements for SPC changes occurred most frequently for a PSUR submitted within 5–10 years of authorization and significantly less frequently for products older than 10 years (*p* < 0.05). With the exception of recombinant blood products, SPC changes were required for products belonging to all mechanistic classes. The proportions of PSURs that resulted in safety-related follow-up requirements were highest for the classes of interferons and monoclonal antibodies and lowest for recombinant blood factors, hormones and growth factors. Changes to the SPC were required most often for products of the ATC class of antineoplastics and immunomodulators when compared to all other ATC classes (*p* = 0.01). No significant differences were found for other ATC classes. Safety-related follow-up requirements occurred throughout the lifecycle of the products. However, the proportion of PSURs that included follow-up requirements to address potential risks was significantly lower for products that were older than 10 years (65 vs. 90 %; *p* < 0.01).

**Table 3:** Subgroup analysis of association between product characteristics and identified safety concerns

Subgroup	SPC variation, *n* (%)	Ratio	95 % Lower limit	95 % Upper limit
Period covered by PSUR				
≤6 months (*n *= 26)	8 (31 %)	1 (reference)		
>6 months - 12 months (*n* = 24)	11 (46 %)	1.49	0.72	3.07
>12 months (*n* = 20)	7 (35 %)	1.14	0.50	2.61
Period between international birth date and date of PSUR				
>10 years (*n* = 20)	3 (15 %)	1 (reference)		
5–10 years (*n* = 26)	14 (54 %)	3.59*	1.19	10.81
0–5 years (*n* = 24)	9 (38 %)	2.50	0.78	8.01
Mechanistic class				
All other products (*n *= 33)	10 (30 %)	1 (reference)		
Monoclonal antibodies (*n* = 19)	11 (58 %)	1.91	1.00	3.64
Hormones (*n* = 18)	5 (28 %)	0.92	0.37	2.27
ATC class				
All other products (*n *= 44)	11 (25 %)	1 (reference)		
Immunomodulators & antineoplastics (*n* = 26)	15 (58 %)	2.31*	1.26	4.24
First in ATC class				
Yes (*n* = 11)	3 (27 %)	1 (reference)		
No (*n* = 59)	23 (39 %)	1.43	0.52	3.95
Biosimilar				
Yes (*n* = 6)^a^	2 (33 %)	1 (reference)		
No (n = 64)	24 (38 %)	1.08	0.33	3.50
Orphan drugs				
Yes (*n* = 8)	2 (25 %)	1 (reference)		
No (*n* = 62)	25 (40 %)	1.61	0.47	5.56
Approved under exceptional circumstances				
Yes (*n *= 7)	1 (14 %)	1 (reference)		
No (*n* = 63)	25 (40 %)	2.78	0.44	17.49

*p < 0.05 (2-sided Fisher’s exact test)

SPC, Summary of Product Characteristics

^a^label changes followed a class review

### Contribution of follow-up requirements in identifying new safety concerns

**Fig. 3:**
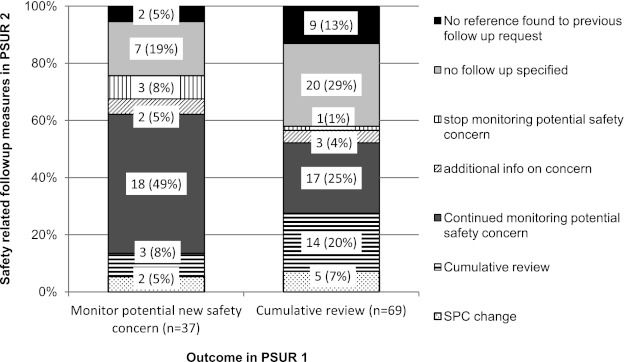
Outcome of new potential safety concerns. Potential safety concerns identified in the first of two consecutive PSURs were defined as either requests for cumulative reviews or the monitoring of potential new safety concerns. The *vertical axis* shows the outcome of the potential safety concern in the following PSUR. *SPC* Summary of Product Characteristics

For 45 products, multiple PSURs were submitted during the study period. In 28 (62 %) of these, potential new safety concerns were identified, amounting to 106 new potential safety-related follow-up requirements. This included 69 requirements for ‘cumulative reviews’ of data on potential safety concerns and 37 requirements for the initiation of ‘monitoring potential new safety concerns’ (Fig. 3). Of these 106 new potential safety concerns, seven (7 %) resulted in SPC changes in the following PSUR, 55 (53 %) resulted in an additional safety-related follow-up requirement and 31 (29 %) required no follow-up. For the remaining 11 (10 %) of the issues addressed, no reference could be identified.

## Discussion

We evaluated the role of PSURs in facilitating authorization communication between regulators and MAHs. Our results show that 37 % of the PSUR assessments of the biopharmaceuticals included in our study concluded that a change in the product labeling was needed. Potential safety concerns continued to emerge throughout the lifecycle of these biopharmaceuticals, and safety-related follow-up measures were required for 83 % of all these products. Despite the fact that new potential safety concerns were identified in the majority of PSURs, a minority (9 %) of these requirements led to regulatory action in the following PSUR.

How does PSUR assessment contribute to finding new safety concerns? PSURs were cited as a contributing source in 40 % of the type II variations in a study of a sample of biopharmaceuticals [9]. Alvarez et al. found that in 64 % of a selection of safety signals, the earliest detection could be traced back to PSURs. The results of our study seem to confirm that PSURs contribute to a significant portion of the post-approval product label changes. However, the majority of the follow-up requirements that addressed these new potential safety concerns did not result in regulatory action, but most resulted in additional follow-up requirement. Of all the safety-related follow-up requirements, 7% resulted in SPC changes indicating a modest additional impact of follow-up requirements to monitor or review specific safety concerns. None of the PSURs included in our analysis identified a shift in the overall B/R balance of the product. It has been noted that PSURs are not intended to be a ‘signal detection tool,’ but rather as a tool to periodically re-evaluate the overall safety profile of a medicinal product [16]. This periodicity ensures a weighing of all the available evidence, but may preclude PSURs from contributing to urgent regulatory actions. On the other hand, if the PSUR assessment process never results in some form of regulatory action, one can question whether PSURs are the most effective regulatory instrument to manage the post-approval safety of medicines.

We did not differentiate between proposals by the MAHs and requirements originating from the assessor at the regulatory agency. However, we did identify a considerable number of PSURs (46; 66 %) with quality issues or with requirements to provide missing data. Thus, the assessment procedure ensures that the task of evaluating the data will be performed meticulously and thoroughly. We did not evaluate if such quality-related follow-up requirements contribute to the initiation of regulatory actions.

Despite the introduction of a harmonized format in 1996, PSURs differ considerably in structure, content and presentation of safety data, which may complicate the assessment procedure. These differences may originate from the fact that different companies have different working methods, but also because regulators require data to be presented or analyzed in different ways. European legislation has been adopted that stipulates changes to pharmacovigilance requirements, including PSUR reporting. For example, detailed information of adverse drug reactions is only to be submitted to the European adverse event database (EudraVigilance). In addition, for generic products and products with well-established therapeutic use, PSURs will no longer be routinely required [8]. Although intended to be summaries of adverse events to facilitate a periodic safety evaluation, many PSURs are long and complicated documents. The new format requires a more concise document that includes a discussion of both the benefits and risks, with an emphasis on identifying changes in the overall B/R balance. Similar proposals have also been released for consultation by the ICH [6]. In addition to the increased emphasis on B/R assessments, the ICH proposes a modular design to facilitate differences in international reporting requirements and a greater harmonization with pre-authorization safety reports. Routine monitoring of the outcome of PSUR assessments may provide valuable information for the evaluation of the impact of these regulatory changes.

In our analysis, the follow-up requirements differed considerably in terms of their level of detail. For example, some safety concerns, described requirements to monitor the broad category of ‘infections’ or ‘malignancies’, while others required the follow-up of the more specific safety concerns ‘progressive multifocal leukoencephalopthy’ or ‘tuberculosis’. This difference posed a challenge in comparing the outcomes of different assessment reports. Therefore, it was decided to use dichotomous (Y/N) variables regardless of the level of detail included. In addition, many assessments lacked uniform structure and terminology. Although this is an inevitable result of differences between individual assessors, it also indicates a lack of European standards and protocols for assessing PSURs. Increased harmonization of the assessment procedure may improve the overall process. The use of harmonized terminology for adverse events using the Medical Dictionary for Regulatory Activities (MedDRA) has facilitated the identification and communication of safety issues [17]. The establishment of accepted terminology for follow-up requirements could further harmonize the PSUR assessment process, which may contribute to the quality of the procedure.

We chose SPC variations as an outcome measure for regulatory action because the SPC is the main risk communication method employed by European regulators and because they could be most reliably identified [18]. This should not be interpreted as if the effectiveness of post-approval safety is reflected only in the proportion of SPC variations—i.e. the absence of a safety concern in the study period does not necessarily signify that regulatory oversight is falling short. Finally, it has been questioned whether product labeling is an effective risk minimization instrument [19, 20]. This question was beyond the scope of the current study.

We found that regulatory actions occurred significantly more often after assessment of PSURs of products belonging to the ATC class of ‘immunomodulators and antineoplastic agents.’ This class contained more products with a unique target (mostly monoclonal antibodies) relative to the class of hormones, for example, which included several products with the same target for which extensive experience had been gained, such as insulins and erythropoietins and several biosimilars. Nevertheless, there may be products that hold a greater risk of post-approval safety concerns, warranting a risk-based approach toward PSUR reporting requirements. This conclusion is in line with the results of a study which reported that a large proportion of post-approval safety warnings issued for biopharmaceuticals are related to their immunomodulatory effects [12].

It has been previously demonstrated that about one-third of post authrorization safety communications concern products which have been authorized for more than 10 years [21]. Our data suggest that PSUR assessments, at least for biopharmaceuticals, lead to safety-related follow-up requirements throughout their lifecycles. Given the fact that the safety profile of newly authorized products is not well established, a carefully designed pharmacovigilance program is particularly important at the early stages of drug authorization [22]. However, for products approved for longer than 10 years, the frequency of SPC changes was significantly lower compared to younger products, supporting reduced PSUR reporting requirements for established products, as proposed by the new European pharmacovigilance legislation. However, our study included 70 PSURs, which limited the ability to demonstrate statistically significant differences between the frequencies of required SPC changes within subgroups.

The PSUR evaluations which we assessed rarely concluded that a safety signal was no longer considered a safety concern. This may eventually lead to great volumes of information that need to be reviewed even in the absence of real safety problems [23]. It is recognized that the ‘demonstration’ of the absence of a safety risk is very challenging. In practice, the assessment procedure is more focused on identifying new potential risks, rather than concluding that there is no evidence to support a previous safety concern.

Our study focused on the assessment of PSURs in Europe. Other regions have been reported to operate differently with regard to the handling and assessment of adverse event information, including PSURs [24]. An analysis of the PSUR assessment procedure in other regions could thus yield different results.

Unlike for biopharmaceuticals, adverse events of small molecules are often related to toxicity and/or overdose. Data on this is usually lacking at the time of authorization and is added to the label through post authorization SPC updates [25]. In addition, unlike the case for biopharmaceuticals, safety events of small molecules are included in a considerable number of post-approval regulatory actions related to toxicity as a result of overdose, data which are often lacking at the time of authorization and which may be included in post-authorization SPC updates [26]. Therefore, it could be that for small molecules the outcome of pharmacovigilance activities differ, and future research could compare our results with those for small molecules.

A major problem when assessing the contribution of PSURs in the safety management of (bio)pharmaceuticals is the lack of a control group. As PSURs are mandatory for all products, it is not possible to determine whether safety findings would also have been identified and managed without them. Furthermore, PSURs are not publicly available, which complicates the assessment of their role in relation to other post-approval activities. Wider access to PSUR data would facilitate future studies into the role of PSUR reporting. In addition, PSURs also serve as a vehicle to monitor and streamline regulatory procedures, such as risk management plans and marketing authorization renewal procedures. We examined the role of PSURs, but their value should be considered in conjunction with all available regulatory instruments.

In conclusion, oversight is clearly needed to ensure that a product’s benefits continue to outweigh its risks, and PSURs facilitate the weighing and monitoring of such events at predetermined time points. As such, PSURs are clearly a vehicle to drive regulatory dialogue, but determination of their contribution to the safe use of medicines as ancillary to existing pharmacovigilance requirements remains a challenge.
